# Real-time Monitoring of Non-specific Toxicity Using a *Saccharomyces cerevisiae* Reporter System

**DOI:** 10.3390/s8106433

**Published:** 2008-10-16

**Authors:** Anna-Liisa Välimaa, Anniina Kivistö, Marko Virta, Matti Karp

**Affiliations:** 1 Department of Chemistry and Bioengineering, Tampere University of Technology, P.O. Box 541, FI- 33101 Tampere, Finland; E-Mails: anniina.kivisto@tut.fi (A.K.); matti.karp@tut.fi (M.K.); 2 Biosensing Competence Centre (BCC), Tampere University of Technology, P.O. Box 541, FI- 33101 Tampere, Finland; E-Mails: anniina.kivisto@tut.fi (A.K.); matti.karp@tut.fi (M.K.); 3 Department of Applied Chemistry and Microbiology Division of Microbiology, P.O. Box 56, FI-00014, University of Helsinki, Finland; E-Mails: marko.virta@helsinki.fi (M.V.)

**Keywords:** Luciferase reporter gene, *Photinus pyralis*, bioluminescence, rapamycin, toxic metal

## Abstract

Baker's yeast, *Saccharomyces cerevisiae*, is the simplest and most well-known representative of eukaryotic cells and thus a convenient model organism for evaluating toxic effects in human cells and tissues. Yeast cell sensors are easy to maintain with short generation times, which makes the analytical method of assessing antifungal toxicity cheap and less-time consuming. In this work, the toxicity of test compounds was assessed in bioassays based on bioluminescence inhibition and on traditional growth inhibition on agar plates. The model organism in both tests was a modified *S. cerevisiae* sensor strain that produces light when provided with D-luciferin in an insect luciferase reporter gene activity assay. The bioluminescence assay showed toxic effects for yeast cell sensor of 5,6-benzo-flavone, rapamycin, nystatin and cycloheximide at concentrations of nM to μM. In addition, arsenic compounds, cadmium chloride, copper sulfate and lead acetate were shown to be potent non-specific inhibitors of the reporter organism described here. The results from a yeast agar diffusion assay correlated with the bioluminescence assay results.

## 1. Introduction

Since the amount of toxic chemicals present in our daily lives is huge and increasing, a sensitive, rapid, robust and cheap toxicity assay is needed for acute toxicity assessment in eukaryotic cells. The topic has been studied a lot and several biological toxicity tests have been developed. Microbial and biochemical tests for assessing chemical toxicity, especially in the aquatic environment, have been reviewed by Bitton [1]. Since that time the methods and several models including insect [2], fish [3], *Daphnia magna* [4] and tumor cell lines [5] have been generated for assessing toxicity of molecules to eukaryotic cells and tissues. None of these alone can answer the demands for a general model which is sensitive, economic and good in predicting the effects of toxic compounds against humans. Model organisms of different species typically have different sensitivities, which means that each biological test differs more or less from others and may not give universal results when assessing toxicity [6]. Additionally, with biological systems, evaluation of toxicity of fat soluble, highly hydrophobic compounds is difficult, since these compounds may be underestimated [7].

Among yeasts, the well-known baker's yeast, *Saccharomyces cerevisiae*, is an excellent organism for evaluating genetic functions of more complex eukaryotic organisms, including toxic effects on human cells and tissues [8]. Like most yeasts, *S. cerevisiae* is easy to cultivate and manipulate genetically [9] and furthermore, inexpensive and available in all grocery stores. On the other hand, even though mammalian cells share functional homologues with the yeasts, some chemicals assayed to be non-toxic for *S. cerevisiae* may be toxic to human cells and tissues. One example of such homologies is an inducible multidrug resistance ABC transporter Pdr5p, which is able to export a broad range of chemically distinct compounds [10]. Therefore, avoiding long assessment time (overnight incubation) will minimize the effects of non-specific inducible efflux pumps that may result in underestimation of acute toxicity.

Previously reported biological acute toxicity assays include bacterial agar plate techniques or assays measuring growth inhibition microscopically or spectrophotometrically [6]. On the other hand, yeast based assays for toxicity studies have been based on changes of culture respiration [6, 11-15], cell growth [16, 17], decreased fluorescence emission by green fluorescence protein (GFP) [18], bioavailability of copper and lead [19], or decrease in bioluminescence in the luciferase reporter gene assay [20]. Genetically modified yeast cells with human regulatory elements have been successfully used also in assessing estrogenicity [7, 21-25] and androgenicity of compounds [26] and detecting cell wall-disturbing agents [27].

In this study we introduce an alternative approach for the assessment of non-specific toxicity of several chemicals, even in real-time. Our toxicity assay is based on *S. cerevisiae* transformed with a modified firefly (*Photinus pyralis*) luciferase gene (*luc*) as a reporter for genetic response. The *luc* gene is inserted into the plasmid pRS316/GPD-PGK between the constitutive promoter GPD and PGK terminator. The plasmid produces light constitutively [21]. Firefly luciferase catalyses the following reaction: Luc + D-luciferin + ATP → oxyluciferin + AMP + CO_2_ + PP*i* + light. The resulting luminescence (yellowish light) can be measured very sensitively in real-time. In our assays, we use D-luciferin substrate at a pH of 5.0, because in the modified firefly luciferase the last three amino acids of the enzyme have been truncated. The natural peroxisomal targeting signal (Ser-Lys-Leu) [28] lacking from the *C*-terminus of the enzyme results in a cytoplasmic expression, which leads to high level of light emission. Under such conditions, where full length firefly luciferase is used [20] D-luciferin must traverse cytoplasmic and peroxisomal membranes to give light emission. This together with a D-luciferin substrate at a pH of 3.0 results in a low level of light emission [20]. We have previously shown that keeping the yeast cells at pH of 5.0 increases the light emission and the growth rate, providing more viable cells for toxicity measurement [29]. Furthermore, an assay can be done in a multi-well plate and the light emission produced by luciferase can be measured simply by adding D-luciferin substrate after an exposure of few hours or even in real-time. We show in this study that the acute toxicity of several model compounds representing completely different kinds of molecular families or structures against eukaryotic organisms can now be performed with light-emitting intact yeast cells on the contrary to Hollis *et al.* [20].

## 2. Results and Discussion

### 2.1 Bioluminescence assay

In this work, we estimated the toxicity of selected chemicals by exposing genetically modified yeast cells and measuring the luminescence produced in the presence of D-luciferin. In our study, the response to different chemicals varied a lot from activation to complete inhibition of light emission depending on the concentrations used. The toxicity of two compounds, 5,6-benzoflavone and rapamycin were monitored continuously in real-time. According to the results (Table 1), the chemicals tested can be divided into toxic or nontoxic for *S. cerevisiae*.

#### 2.1.1 Antimicrobial agents

**Figure 1.**
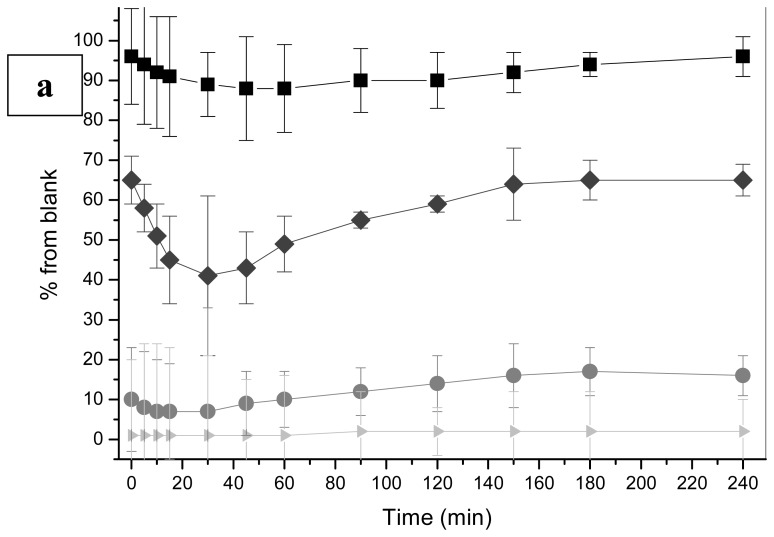

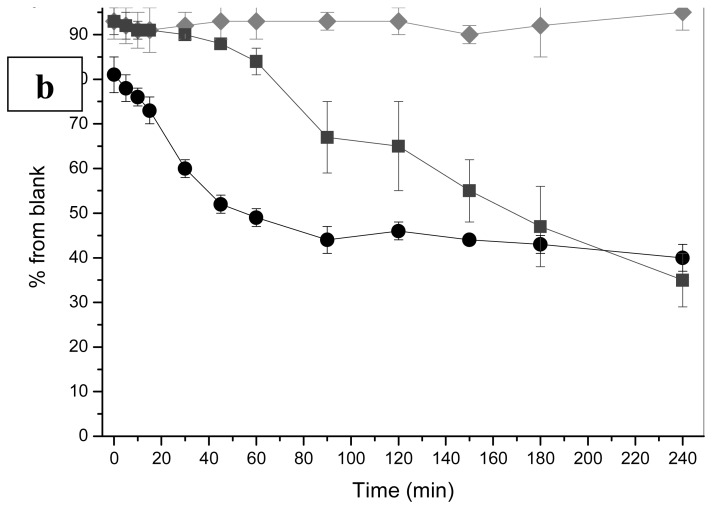
**a.** Bioluminescence response to 5,6-benzoflavone in real-time monitoring during exposure of 4 h. Squares (0.75 nM), diamonds (7.5 nM), circles (75 nM) and triangles (750 nM) denote for concentration of 5,6-benzoflavone used, respectively. The error bars are shown for triplicate parallel measurements. **b.** Bioluminescence response to rapamycin in real-time monitoring during an exposure of 4h. Rapamycin reveals totally different bioluminescence response. The error bars are shown for triplicate parallel measurements and the symbols are diamonds (0.05 μM), Squares (0.5 μM), and circles (5 μM) denote for concentration of rapamycin used, respectively.

Evidently, 5,6-benzoflavone (Table 1) is the most potent among the chemicals tested and concidered as toxic for yeast cells. Additionally, a concentration of 750 nM caused total inhibition of bioluminescence throughout an exposure of 4 hours and at 7.5 nM the bioluminescence response, depending on the exposure time, varied between 41% (after 30 min exposure) of the response in blank to 65% of the response in blank (Figure 1a). The results are not unexpected, since 5,6-benzoflavone is known to be a strong inducer of certain enzymes belonging to the CYP 450 superfamily and has the same induction potency as the carcinogenic benzo(a)pyrene. For this reason 5,6-benzoflavone is widely used for studies in toxic effects in mammals mediated by aryl hydrocarbon receptor [30]. On the other hand as far as we know, no data in the literature on non-specific toxicity measured by a yeast-based bioassay is available for 5,6-benzoflavone.

We also obtained interesting results in an exposure of rapamycin (shown in Figure 1b as an example). It is clearly evident that the toxicity of 5,6-benzoflavone (Figure 1a) and rapamycin (Figure 1b) can be followed in real-time after the administration of the toxicant and the luciferase substrate D-luciferin. The emission is dose-dependent and the toxicity can be followed kinetically inside the thermostated measurement chamber of the multilabel reader.

**Figure 2.**
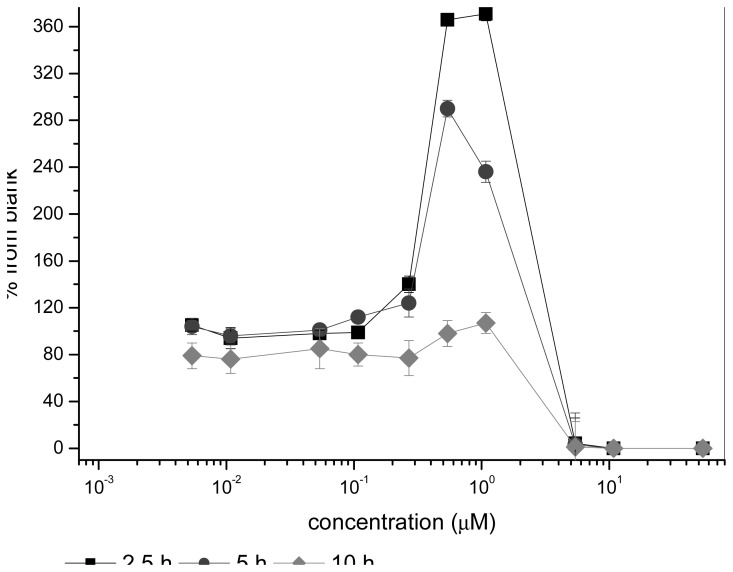
The bioluminescence response to nystatin, exposure of 2.5, 5 and 10 h. Squares (2.5 h), circles (5 h) and diamonds (10 h) denote for exposure used, respectively. The error bars are shown for triplicate parallel measurements.

The bioluminescence responses to nystatin (Figure 2) were different from the responses to other tested chemicals. Low concentrations of nystatin had no inhibitory effect on the yeast sensor cells; the light production was similar to that of the blank (100%). At a concentration of 0.54-1.08 μM, the bioluminescence response, depending on the exposure time, varied between 290% (an exposure of 5 hours) and 371% (an exposure of 2.5h), while a concentration of 5.40 μM caused total inhibition of bioluminescence and can be considered as toxic for the yeast sensor. After an exposure of 10 hours, the peak values at low concentrations of nystatin were not visible any more. This effect was seen repeatedly and was statistically significant in the control experiments. One explanation for this odd behaviour could be due to nonspesific, uncharacterized inhibition of metabolic pathways leaving more ATP to be used for the luciferase reaction. Thus, the phenomenom may be explained by mechanism of nystatin. This antifungal agent interacts with membrane sterols and damages cell membranes thus causing the leakage of intracellular K^+^ and increased permeability to protons [32]. Maybe excess of protons are used by vacuolar membrane H^+^-ATPases [33] to generate ATP which can be seen as a peak in a narrow concentration area. Similar high peak of ATP concentration has been observed in yeast cells during the lag growth phase, while in the logarithmic growth phase the amount of ATP remained constant being 2 μmol ATP/g yeast [34]. Another explanation would be minor increases in transcription of specific genes related to stress responses or modes of action as described for bacteria [35, 36]. The luminescence peak has also been characterized previously in bacterial reporter gene systems [37].

Sodium dodecyl sulfate (SDS) is an anionic detergent (surfactant) that is commonly used in household detergents and in various industrial and academic applications. In our studies, a concentration of 133.5 mM caused a 50% decrease in luminescence after a 2.5 h of exposure, while after a 5 h of exposure the same effect was seen at a concentration of 69.5 mM (Table 1). The increase in toxicity may be due to a prolonged exposure. SDS has more time to damage membrane structure and solubilizes proteins. [38].

Our results contradict those by Sirisattha *et al.* [38], where exposing yeast cells to 0.01% (34.7 mM) SDS for 2 h caused damages to membrane structure, altered carbon metabolism and induced oxidative stress in yeast cells. The difference could be partially attributed to different assay methods or experimental conditions. Our toxicity assay is based on *S. cerevisiae* transformed with a modified firefly luciferase gene as a reporter for genetic response. The bioluminescence produced by luciferase can be measured in intact living cells by adding the D-luciferin substrate after an exposure. In their studies, Sirisattha *et al.* used microarrays monitoring gene expressions. The yeast strain (also different from ours) was cultivated in a different medium and total RNA was extracted. Additionally, the incubation temperatures differed.

Interesting and unexpected results (Table 1) were also obtained by exposing the yeast sensor cells to cycloheximide (a protein synthesis inhibitor). An exposure of the yeast sensor cells to ths compound for 2.5 h had no toxic effects, while an exposure of 5 h was highly toxic. At the beginning of incubation, a MFS efflux pump can remove the drug from its cellular targets to vacuoles resulting in increased resistance [39], while under a prolonged exposure the vacuolar capacity is insufficient in preventing cycloheximide to concentrate in a cytosol. Additionally, beside the MFS efflux pump, cycloheximide resistance is induced by an efflux pump belonging to the ABC superfamily. Possibly, the latter efflux pump like Pdr5p is strongly expressed during the exponential growth phase, whereas during the stationary growth phase or limitations of important nutrients the expression level of the efflux pump rapidly decreases [40] thereby, making the yeast cells more susceptible to cycloheximide.

The results show that at least 1000-fold increase in toxicity with increasing exposure time was observed for cycloheximide (the highest concentration tested was 0.43 mM). Ten or more-fold increase in toxicity with increasing exposure time was observed for rapamycin, arsenic(V)oxide, sodium-*m*-arsenite and cadmium(II)chloride, whereas 5,6-benzoflavone, ketoconazole, nystatin, sodium azide and SDS showed no increase (Table 1, paragraph 2.2). An increase in sensitivity after a 5 hours exposure time may be due to changes during the growth phase of the sensor yeast. One of the most important changes concerning the drug resistance is probably the altered expression of multidrug efflux pumps or protein transporters belonging to the ABC transporters or members of the major facilitator superfamily (MFS) that can transfer a wide range of chemically dissimilar compounds with different affinity to the extracellular space thus conferring the pleiotropic (or multidrug) drug resistance (PDR) [41]. Some of these efflux pumps are plasma membrane proteins like the most studied Pdr5p. Moreover, a vacuolar membrane efflux pump such as the glutathione S-conjugate transporter that confers resistance to arsenite, cadmium and lead is known [42]. Efflux pumps are noticed to be activated in an early stage by external stress. For example, Pdr5p was induced only after a 4 minutes of exposure to benomyl [43] and was strongly expressed during the exponential growth phase, while during the stationary growth phase Pdr5p levels decreased rapidly. Additionally, Pdr5p needs glucose for functioning and the limitation of glucose or other important nutrients such as nitrogen reduces Pdr5p expression [40].

#### 2.1.2 Heavy metals

Table 1 shows that even at the millimolar concentrations the heavy metals such as arsenic compounds, cadmium chloride and lead acetate had little if any non-specific toxicity to *S. cerevisiae*, although toxic effects were shown to increase slightly with increasing exposure time at the tested concentrations. These results were expected, as *S. cerevisiae* has been known to have mechanisms for detoxification and heavy metal resistance: efflux that is an active extrusion of the heavy-metal ion from the cell, accumulation into vacuoles as glutathione conjugates and reduction to a less toxic oxidation state [44]. To produce the resistance to arsenite, *S. cerevisiae* has two independent transport systems: a plasma membrane transporter and a vacuolar membrane transporter that belongs to the ABC transporter superfamily. Thus arsenite ions are extruded from a cytosol or accumulated into vacuoles as glutathione conjugates that require glutathione as a reductant [45]. Moreover, resistance to cadmium and lead is due to the same glutathione S-conjugate transporter [42].

In our experiment, trace elements, such as copper sulfate, zinc- and magnesium- chloride were little or non toxic to *S. cerevisiae* and zinc- and magnesium- chloride even increased the signal intensity and improved the health of the yeast cells at the tested concentrations. This was expected, because Cu and Zn are biologically essential trace elements and a component in a variety of enzymes and DNA-binding proteins [42].

### 2.2 Agar diffusion assay compared to bioluminescence assay

To compare and validate the results from the bioluminescence assay, a conventional agar diffusion assay (ADA) was performed. Toxicity of the selected chemicals was estimated by spotting the test sample in filter papers (Ø 6 mm) on the surface of the agar plates containing logarithmic grown yeast sensor strain and then measuring the growth inhibition zones after overnight incubation at 30 °C. The lowest chemical concentration at which an inhibition zone could be observed was defined as a value of minimal inhibitory concentration (MIC).

**Figure 3.**
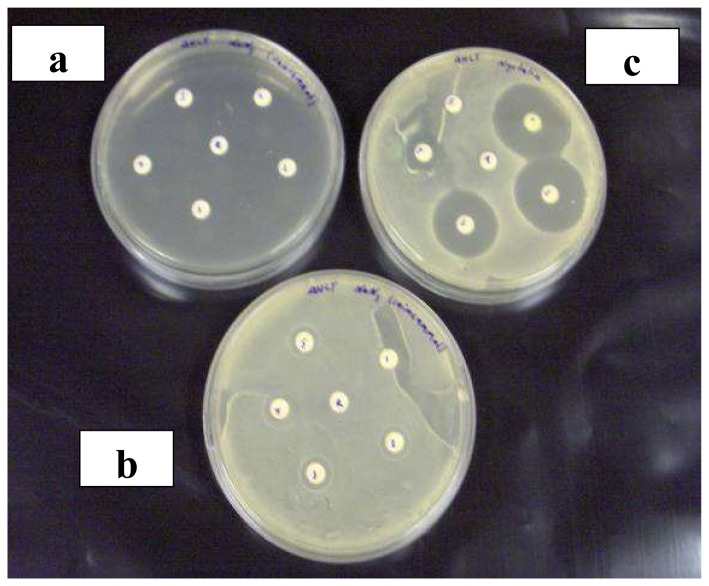
Toxicity assay on agar plates. A plate containing stronger sodium azide solution (a) has no visible growth, whereas in one containing more dilute sodium azide solutions (b) no clear inhibition circles are visible. A plate containing nystatin solutions (c) is a good example of toxicity assay with clear inhibition zones.

The results (Table 1 and Figure 3) showed correlation with the results from the bioluminescence assay. In general, the toxic chemicals showed toxicity in the ADA, whereas the ones predicted to be nontoxic had no toxic effects. Ketoconazole was an exception showing toxic effects to the yeast sensor strain in the ADA but having no significant response in the bioluminescence assay. To improve solubility ketoconazole was dissolved into acetate buffer (pH 3.6). However, better solubility does not necessarily mean increased bioavailability. Ketoconazole is an antifungal drug that contains imidazole and piperazine parts. For cellular uptake they both should be in a nonprotonated form which happens in a neutral environment [46]. Maximal uptake of ketoconazole by the human pathogenic yeast *Candida albicans* occurs between pH 6.5-7, while in an acidic environment ketoconazole penetrates into yeasts poorly. It has been suggested that even 200-fold increase in sensitivity towards *Candida albicans* has been reached as pH increases from 3 to 5 [47]. Thus, ketokonazole having no significant response in the bioluminescence assay could be partly explained by a low pH 3.6 of the solvent used. The other explanation might be an expression of PDR pumps induced by weak acid stress. During the normal yeast metabolism pH of as low as 3 (data not shown) is achieved because of acetate production. This weak acid stress induces certain PDR pump that extrudes acetate from yeast cells thereby preventing penetration of external acetate [41]. However, in the ADA the inhibitory effect of ketoconazole was observed. This could be attributed to longer incubation time (1-3 days) compared to the bioluminescence assay. The yeasts are in the late stationary phase, in which the expression level of efflux pumps decreases [40] thereby making the yeast cells more susceptible to ketoconazole.

Concerning the bioluminescence assay, sodium azide was highly toxic (Table 1), however, no visible growth was detected in the ADA throughout the plate containing the highest sodium azide concentration (Figure 3) and moreover, no clear inhibition zones in the plate containing more dilute sodium azide solutions (Figure 3) are visible. These observations indicate that sodium azide with molecular weight of 65.01 g/mol is such a small molecule that it is assumed to diffuse equally into agar, inhibiting growth totally. As we have shown here, a traditional toxicity assay on agar plates is not a convenient method in measuring toxicity of very small molecules in a yeast toxicity assessment.

## 3. Experimental Section

### 3.1 Chemicals

Zinc(II)chloride (ZnCl_2_), cadmium(II)chloride (CdCl_2_)_,_ lead(II)acetate [(CH_3_COO)_2_Pb], sodium azide (NaN_3_) and 30% hydrogen peroxide were from Merck & Co (Whitehouse Station, NJ, USA), sodium-*m*-arsenite (NaAsO_2_), nystatin (C_47_H_75_NO_17_), sodium dodecyl sulfate (SDS, C_16_H_25_OSO_3_Na) and 5,6-benzoflavone (B-NF, C_2_H_6_OS), dimethyl sulfoxide (DMSO), trisodium citrate, adenine hemisulfate salt, l-leucine, l-histidine and l-tryptophan from Sigma-Aldrich Co (St. Louis, MO, USA), Copper(I)sulfate (CuSO_4_) was from VWR International (West Chester, PA, USA), magnesium(II)chloride (MgCl_2_) from Mallinckrodt Baker (Phillipsburg, NJ, USA), arsenic(V)oxide (As_2_O_2_) and citric acid (Fluka) from Sigma-Aldrich Co (St Louis, MO, USA) and cycloheximide from MP Biomedicals (Aurora, OH, USA). Ethanol was from Altia Plc (Helsinki, Finland), antibiotic assay disks of diameter of 6 mm were from Schleicher & Schuell (Keene, NJ, USA). Yeast nitrogen base w/o amino acids and agar-agar (Difco) were purchased from Voigt Global Distribution (Lawrence, KS, USA) and D-Luciferin was supplied by Biothema AB (Handen, Sweden).

### 3.2 Sample preparation

Stock solution from each test chemical was prepared by dissolving the solid chemical into the following solvent: ketoconazole was dissolved into acetate buffer (pH 3.6) nystatin and B-NF into DMSO. Other chemicals were dissolved into sterile deionized water. Sample sets were prepared by diluting stock solutions in a ratio of 1:10 with the respective solvent. Rapamycin solutions were stored at -20°C and CdCl_2_, NaN_3_, As_2_O_2_, NaAsO_2_, cycloheximide, nystatin, ketoconazole and B-NF solutions were stored at +4°C. Other samples were preserved at room temperature and protected from light.

### 3.3 Yeast strain cultivation

The yeast strain used in this study was *S. cerevisiae* transformed with a modified firefly (*Photinus pyralis*) luciferase gene (*luc*) inserted in the plasmid as a reporter for genetic response, BMA64-1A+pRS316luc [29]. The insertion into this plasmid facilitates the incorporation of the reporter gene into the chromosome of *S. cerevisiae*. Firefly luciferase catalyses the following reaction: Luc + D-luciferin +ATP → oxyluciferin + AMP + CO_2_ +PP*i* + light. The resulting luminescence (yellowish light) can be measured very sensitively and in our assay simply by adding D-luciferin substrate after an exposure of few hours or even in real-time. We use the D-luciferin substrate at pH of 5.0, because in the modified firefly luciferase the last three amino acids of the enzyme have been truncated. The natural peroxisomal targeting signal (Ser-Lys-Leu) [28] lacking from *C*-terminus of the enzyme results in cytoplasmic expression, which leads to high levels of light emission.

For both bioluminescence and conventional agar diffusion assay, *S. cerevisiae* was cultivated overnight at 30°C and 300rpm shaking in liquid synthetic dextrose medium (SD) that contains yeast nitrogen base w/o amino acids (6.7 g/L) and D-glucose 40 mL/L of a 40% (w/v) supplemented with adenine (5 g/L) and the essential amino acids *l*-histidine (2 g/L), *l*-leucine (10 g/L) and *l*-tryptophan (2 g/L). Yeast nitrogen base w/o amino acids and D-glucose were autoclaved (121°/15 min) and adenine and amino acids were filter sterilized (pore size 0.2 μM). pH of the SD medium was checked time to time and pH was 4.8±0.2. For the bioluminescence assay, the yeast cells were grown to an optical density (OD_600_) of about 3.0 and after that the culture was diluted with the same medium to OD_600_ of 0.8 and right after were used in the assay. For agar diffusion assay, the same medium described above was solidified with agar-agar.

### 3.4 Assay procedures

#### 3.4.1 Bioluminescence assay

Ten or 1 μL aliquots of the sample sets and reference samples (blank solvent) were pipetted in triplicate into a white 96-well plate (Thermo Electron Corporation, Finland) with hydrogen peroxide (0.3% or 3%, depending on the test) as a positive control. Ninety or 100 μL aliquots of the diluted yeast culture were then added into the wells containing samples. In the wells, the final amount of water or acetate buffer was 10 % and DMSO 1%.

Plates were then incubated at 30°C and 300 rpm for 2.5 h or 5 h. After the incubation, 100 μL aliquots of 1 mM D-luciferin (in 0.1 M Na-citrate buffer pH 5) were pipetted into the wells and the bioluminescence was immediately measured with a Chameleon Multilabel Detection Platform (Hidex Oy, Turku, Finland). Furthermore, for real-time monitoring, 5,6-benzoflavone and rapamycin were measured continuosly (Figure 1) and light emission level of the blank, expressed as relative light units (RLU) was set as 100%. The IC_50_ of all tested chemicals was calculated. Because the bioluminescence response curves of nystatin were different from the curves of other tested chemicals, an exposure time of 10 hours was used to see if the same phenomenon could be seen under a prolonged incubation time.

#### 3.4.2 Agar diffusion assay

To compare and validate the results from the bioluminescence assay, a conventional agar diffusion assay (ADA) was performed. Aliquots of test sample (five dilutions) and reference solutions were pipetted onto filter papers (Ø 6 mm) which were then spotted on the surface of the SD –agar plates containing the logarithmic overnight grown yeast sensor strain. After overnight incubation at 30 °C, the growth inhibition zones were measured. The lowest chemical concentration at which an inhibition zone could be observed was defined as a value of minimal inhibitory concentration (MIC).

## 4. Conclusions

The goal of this study was to show that our toxicity assay based on a representative of eukaryotic cells *S. cerevisiae* transformed with a modified firefly *(Photinus pyralis)* luciferase gene *(luc)* inserted in the plasmid as a reporter is an alternative method in screening non-specific toxicity of several chemicals in real-time. The toxicity of the test compounds was assessed in the bioassays based on bioluminescence inhibition. The traditional growth inhibition on an agar plate was performed to compare and validate the results from the bioluminescence assay.

It naturally needs to be emphasized that when toxicity testing is concerned, all systems have their advantages and disadvantages. Even though the results from the agar diffusion assay (ADA) correlated with the results from the bioluminescence assay, the ADA has many disadvantages. Making plates for each concentrations and each chemical is time-consuming, and the results can be read only after 1-3 days, whereas in the bioluminescence assay different concentrations of different chemicals can be performed in a multi-well plate and the luciferase activity can be measured from intact living yeast cells even in real-time just by adding D-luciferin. Furthermore, since the ADA reveals only a level of the toxicity, the bioluminescence assay can give some ideas about the character of the toxicity like during 5,6-benzoflavone, rapamycin and nystatin monitoring. Thus, because of the advantages mentioned above and due to the fact that *S. cerevisiae* is easy to cultivate in a pour medium with short generation time, the bioluminescence assay could be a fast and inexpensive tool suitable for toxicity assessments.

## Figures and Tables

**Table 1.** The IC_50_ values (μmol/L) of the chemicals measured by the bioluminescence assay having the exposure of 2.5 h or 5 h compared to the results (minimum inhibitory concentration, μmol/L) of the conventional agar diffusion assay.

**Compound**	**Bioluminescence assay IC50 (μmol/L)**		**Agar diffusion assay**

	**Exposure time 2.5 h**	**Exposure time 5 h**	**Minimum inhibitory concentration (MIC) (μmol/L)**

Antimicrobial agents			

5,6-benzoflavone	0.008	0.006	0.075
Cycloheximide	NT^a^	0.7	42.7
Ketoconazole	NT	NT	1.0
Nystatin	1.6	1.4	54.0
Rapamycin	2.8	0.03	0.2
Sodium azide	25.8	27.4	- b
Sodium dodecyl sulfate	133.5*103	69.5*103	1.8*103



Metals			

Arsenic(V)oxide	2.4*103	0.4*103	85.5*103
Sodium-*m*-arsenite	25.5*103	0.5*103	14.5*103
Cadmium(II)chloride	19.3*103	2.0*103	124.6*103
Copper(I)sulfate	5.1*103	ND	9.8*103
Lead(II)acetate	71.9*103	18.4*103	- c
Magnesium(II)chloride	NT	NA^d^	2060.5*103
Zinc(II)chloride	NT	NA	10265.6*103

a NT, not toxic, the IC_50_–level not reached,

b no growth visible at the assay plate,

c he sample precipitated on the plate,

d NA, not assayed
